# MiR-30-Regulated Autophagy Mediates Angiotensin II-Induced Myocardial Hypertrophy

**DOI:** 10.1371/journal.pone.0053950

**Published:** 2013-01-09

**Authors:** Wei Pan, Yun Zhong, Chuanfang Cheng, Benrong Liu, Li Wang, Aiqun Li, Longgen Xiong, Shiming Liu

**Affiliations:** Cardiovascular Department, the Second Hospital Affiliated to Guangzhou Medical University, Guangzhou Institute of Cardiovascular Disease, Guangzhou, Guangdong Province, China; University of Western Ontario, Canada

## Abstract

Dysregulated autophagy may lead to the development of disease. Role of autophagy and the diagnostic potential of microRNAs that regulate the autophagy in cardiac hypertrophy have not been evaluated. A rat model of cardiac hypertrophy was established using transverse abdominal aortic constriction (operation group). Cardiomyocyte autophagy was enhanced in rats from the operation group, compared with those in the sham operation group. Moreover, the operation group showed up-regulation of *beclin-1* (an autophagy-related gene), and down-regulation of miR-30 in cardiac tissue. The effects of inhibition and over-expression of the *beclin-1* gene on the expression of hypertrophy-related genes and on autophagy were assessed. Angiotensin II-induced myocardial hypertrophy was found to be mediated by over-expression of the *beclin-1* gene. A dual luciferase reporter assay confirmed that *beclin-1* was a target gene of miR-30a. miR-30a induced alterations in *beclin-1* gene expression and autophagy in cardiomyocytes. Treatment of cardiomyocytes with miR-30a mimic attenuated the Angiotensin II-induced up-regulation of hypertrophy-related genes and decreased in the cardiomyocyte surface area. Conversely, treatment with miR-30a inhibitor enhanced the up-regulation of hypertrophy-related genes and increased the surface area of cardiomyocytes induced by Angiotensin II. In addition, circulating miR-30 was elevated in patients with left ventricular hypertrophy, and circulating miR-30 was positively associated with left ventricular wall thickness. Collectively, these above-mentioned results suggest that Angiotensin II induces down-regulation of miR-30 in cardiomyocytes, which in turn promotes myocardial hypertrophy through excessive autophagy. Circulating miR-30 may be an important marker for the diagnosis of left ventricular hypertrophy.

## Introduction

Myocardial hypertrophy induced by angiotensin II (Ang II) is an important cause of cardiac remodeling. Identification of the mechanisms through which Ang II induces myocardial hypertrophy would reveal novel targets for the development of new therapies for cardiac remodeling [1]. The development of myocardial hypertrophy is induced by the synthesis of cardiac contractile proteins, and this is increased by Ang II which stimulates anabolic pathways [2]. Homeostasis in cardiomyocytes is maintained by physiological autophagy, which not only removes misfolded or aggregated proteins, but also plays an important role in the clearance of damaged organelles, such as mitochondria and endoplasmic reticulum [3]. Autophagy may be activated by aberrant protein aggregation to help remove aggregates. However, excessive or deficient autophagy may lead to the development of disease. Cardiomyocyte physiological autophagy is an adaptive and self-protective mechanism that occurs during cardiac remodeling, but excessive autophagy may lead to cardiomyocyte death [4].

Beclin-1, a mammalian orthologue of yeast Atg6, was the first mammalian autophagy-related protein to be identified. Beclin-1 plays an important role in regulating vacuolar sorting protein 34 (Vps-34, a class III phosphatidylinositol-3 kinase), and advances the formation of beclin-1-Vps34-Vps15 core complexes, which induce autophagy [5]–[8]. Previous studies have shown that miR-30a could negatively regulate *beclin-1* gene expression, resulting in decreased autophagic activity in cancer cells such as T98G, MDA-MB-468 and H1299 cells [9]. However, it is not known whether miR-30a can influence autophagy in cardiomyocytes through regulation of *beclin-1* gene expression, and whether excessive autophagy mediates the actions of Ang II to cause myocardial hypertrophy. We hypothesize that myocardial hypertrophy induced by Ang II is mediated by excessive autophagy. The results of our study indicate that Ang II excessively up-regulates cardiomyocyte autophagy by decreasing miR-30 expression, and that this excessive autophagy promotes the development of myocardial hypertrophy. Consistent with this, the expression level of miR-30 in the plasma of peripheral blood was elevated in patients with left ventricular hypertrophy (LVH).

## Materials and Methods

### Animal Models, Echocardiography, and Tissue Staining

Wistar rats were divided randomly into 2 groups:Sham group(n = 6) and transverse abdominal aortic constriction (TAAC) group (n = 8). Rats in the Sham group only underwent exposure of the aorta. In the TAAC group, transverse abdominal aortic constriction was performed between the abdominal aorta and anterior mesenteric artery, until the diameter of the aorta was 0.5 mm. 4 weeks after the operation, ventricular wall thickness and heart chamber size were measured using echocardiography (Philips ie 33, Netherlands). Blood samples were collected from the central veins, and the derived plasma was stored at -80°C. Sample of the heart were taken and fixed with 4% formaldehyde for subsequent staining with hematoxylin-eosin (HE) and Masson’s. Additional heart samples were kept in liquid nitrogen for subsequent RNA and protein isolation. All animal experiments were approved by the Animal Care and Ethics Committee of Guangzhou Medical University.

### The Construction of Plasmids

Coding sequence (CDS) fragments of *beclin-1* were amplified from rat cDNA. Using HindIII and XbaI sites, the *beclin-1* sequence was cloned into a pRc/CMV2 vector (Invitrogen) and identified by DNA sequencing. The over-expression vector, pRc/CMV2-beclin-1, was then constructed. The 3′ untranslated region (3′-UTR) of *beclin-1* and a mutation sequence were amplified from cDNA by fusion PCR. The 3′-UTR and mutation sequence were inserted, respectively, into the HindIII and XbaI sites of a pGL3-control vector (Promega) and identified by DNA sequencing. Therefore, a wild type plasmid was generated containing the 3′-UTR of *beclin-1* (pGL3-*Beclin-1* 3′-UTR-Wild Type), and a mutant plasmid was created containing the mutation sequence (pGL3- *Beclin-1* 3′-UTR-Mutant). The pRL-TK vector (Promega) was used as an endogenous control.

### Culture of Neonatal Ventricular Cardiomyocytes and Interventions Used

Rat ventricular myocytes were isolated by enzymatic digestion of 1- to 3-day-old neonatal rats, as described previously [10]. The intervention measures used were as follows: stimulation with 1 µmol/L Ang II or 10 mmol/L 3-methyladenine (3-MA, an autophagy inhibitor; Sigma); transduction with lentivirus (GenePharma, China), containing beclin-1-specific siRNA, control-siRNA, miR-30a mimic, miR-30a inhibitor, or control-miRNA (Sequences are shown in Table 1). 4×10^5^ of cardiomyocytes were transduced with 20 µl virus (10^9^TU/ml) and polybrene(at a final concentration of 5 µg/ml). The transfection of pRc/CMV2-beclin-1 was performed by using Lipofectamine™ LTX and PLUS Reagents (Invitrogen) according to the manufacturer’s instruction. Cells were stained with 50 µmol/L monodansylcadaverine (MDC, a specific marker for autophagic vacuoles; Sigma) in day 3 after transfection or treatment with drugs.

**Table 1:** primer and small RNA sequences.

Designation	Sequences	
18s		Forward: 5′-ACCGCAGCTAGGAATAATGGA-3′,Reverse: 5′-GCCTCAGTTCCGAAAACCA-3′
ANP		Forward: 5′-GGGGGTAGGATTGACAGGAT-3′Reverse: 5′-CTCCAGGAGGGTATTCACCA-3′
β-MHC		Forward: 5′-CCTCGCAATATCAAGGGAAA-3′Reverse: 5′-TACAGGTGCATCAGCTCCAG-3′
*Beclin-1*		Forward: 5′- AAGATTGAAGACACTGGAGGCA-3′Reverse: 5′- GAGGACACCCAAGCAAGACC -3′
U6		Forward: 5′-GCTTCGGCAGCACATATACTAAAAT-3′Reverse: 5′-CGCTTCACGAATTTGCGTGTCAT-3′
hsa-miR-30a		Forward: 5′-ACACTCCAGCTGGGTGTAAACATCCTCGACTG-3′Reverse: 5′-CTCAACTGGTGTCGTGGA-3′
cel-miR-39		Forward: 5′-ACACTCCAGCTGGGTCACCGGGTGTAAATCAGCT-3′Reverse: 5′-CTCAACTGGTGTCGTGGA-3′
Negative control of *beclin-1*-specific siRNA		Sense: 5′- UUCUCCGAACGUGUCACGUTT-3′Antisense: 5′- ACGUGACACGUUCGGAGAATT-3′
*Beclin-1*-specific siRNA		Sense: 5′- GGACACUCAGCUCAAUGUUTT-3′Antisense: 5′- AACAUUGAGCUGAGUGUCCTT-3′
Negative control of miR-30a mimic		Sense: 5′- UUCUCCGAACGUGUCACGUTT-3′Antisense: 5′- ACGUGACACGUUCGGAGAATT-3′
miR-30a mimic		Sense: 5′- UGUAAACAUCCUCGACUGGAAG-3′Antisense: 5′- UCCAGUCGAGGAUGUUUACAUU-3′
Negative control of miR-30a inhibitor		5′- CAGUACUUUUGUGUAGUACAA-3′
miR-30a inhibitor		5′- CUUCCAGUCGAGGAUGUUUACA-3′

### Dual Luciferase Reporter Assay

Neonatal rat cardiomyocytes were cultured in a 24-well plate. On the 5th day, cardiomyocytes were transduced with lentivirus containing miR-30a mimic and miR-30a inhibitor. 24h after the transduction, pGL3-Beclin-1 3′-UTR-Wild Type (200 ng) or pGL3-Beclin-1 3′-UTR-Mutant (200 ng) was co-transfected with pRL-TK vector (200 ng) into the cardiomyocytes using Lipofectamine™ LTX and PLUS Reagents. Cardiomyocytes were lysised and the relative luciferase activity was measured with the Dual-Luciferase Report Assay System (Promega) on a GLOMAXTM96 microplate luminometer (Promega, USA).

### Real-time RT-PCR

Total RNA was isolated using TRIzol Reagent (Invitrogen). Reverse transcription was performed at 50°C for 50 min, and 85°C for 5 min, using the SuperScript™ III First-Strand Synthesis SuperMix for qRT-PCR kit (Invitrogen). Real-time PCR was performed using the Platinum® SYBR® Green qPCR SuperMix-UDG kit (Invitrogen), with a 7500 Real-Time PCR System from Applied Biosystems. The relative expression of mRNA was calculated using the 2^−△△Ct^ method, and normalized to the expression of 18S (Primer sequences are shown in Table 1).

### Western Blotting

Protein was extracted from cardiac tissue and cardiomyocytes. Equal amounts of protein (50 µg) were separated by SDS-PAGE (150 V, 1 h) and transferred onto a polyvinylidene difluoride membrane (100 V, GAPDH and beclin-1 1.5 h, LC3 40 min). After blocking, the blots were probed with either anti-beclin-1 antibody, anti-LC3 antibody or anti-GAPDH antibody (Cell Signaling Technology) at 4°C overnight. After washing, the membrane was incubated with horseradish peroxidase (HRP)-conjugated AffiniPure rabbit anti-goat IgG(H+L) (EarthOx, USA) for 1 h. After washing again, immunological complexes were detected using the SuperSignal@ West Pico Chemiluminescent Substrate (Thermo Scientific, USA).

### Flow Cytometry

After incubation with MDC at 37°C in the dark for 1 h, the cardiomyocytes were digested with trypsin. The percentage of autophagic vacuoles was detected using MoFlo flow cytometry (355 nm excitation, 525 nm emission; Beckman Coulter, USA) and analyzed with Kaluza analysis software.

### Electron Microscopy

Electron microscopy [11] was performed on a Tacnai 12 Spirit Twin transmission electron microscope, at a magnification of ×13500. Quantitative analysis of autophagosomes was carried out using 10 images from different fields, with the investigator blinded as to the origin of each image. Autophagosomes or autolysosomes were identified by the characteristic structure of a double- or multi-lamellar smooth membrane completely surrounding compressed mitochondria, or membrane-bound electron-dense material.

### Confocal Microscopy

After removing the culture media, the samples were fixed with 3.7% formaldehyde at 37°C. The samples were permeabilized with 1% Triton X in PBST (Triton X: phosphate buffered saline = 1∶100, volume ratio) for 15 minutes. The samples were then incubated with Alexa Fluor®555 Phalloidin (1∶40, Invitrogen) for 20 minutes. The cells were stained with the chromatin dye, DAPI (300 nmol/L, Invitrogen), for 5 min. Images were obtained using a Zeiss LSM 710 confocal microscope (Carl Zeiss, Germany).

### Detection of miRNA in Cardiac Tissue

The miRNA contents of cardiac tissue and cardiomyocytes were detected using the Hairpin-it™ miRNAs qPCR Quantitation Kit (GenePharma, China). The optimal reverse transcription reaction system was: 40 U/µL RNaseOUT 0.125 µL, 10X RT Buffer 2 µL, 10 mmol/L dNTPmix 0.75 µL, 1 µmol/L miRNA specific RT primer 1.2 µL, 200 U/µL MMLV reverse transcriptase 0.1 µL, total RNA 160 ng; DEPC-treated water was added to give a final reaction volume of 20 µL. The reaction conditions used were: 16°C for 30 min, 42°C for 30 min, and 85°C for 10 min. The optimal real-time PCR reaction system was: 2× Real-time PCR Master Mix 10 µL, 5 µmol/L miRNA specific primer set 0.36 µL, 5 U/µL rTaq DNA polymerase 0.2 µL, 25 µmol/L ROX reference dye 0.8 µL, reverse transcription cDNA 2 µL; DEPC-treated water was added to give a final reaction volume of 20 µL. The real-time PCR reaction conditions used were: 95°C for 3 min, followed by 40 cycles of 95°C for 12 sec and 62°C for 50 sec. The expression of miRNA relative to the U6 endogenous control was calculated using the 2^−△△Ct^ method (Primer sequences are shown in Table 1).

### Detection of miRNA in Plasma

A 3 mL volume of whole blood was collected into a tube containing EDTA, and the plasma was obtained by centrifugation. Total RNA was isolated from the plasma using 1.2 mL TRIzol® LS Reagent (Invitrogen). 8 µL total RNA was dissolved in 2.5 µL DEPC-treated water, maintained at 85°C for 5 min. The optimal reverse transcription reaction system and reaction conditions used were: dissolved total RNA 10.5 µL, 10 mM dNTP 2 µL (Promega), RNasin® Ribonuclease Inhibitor 0.5 µL (Promega), miR-30a reverse transcription primer 0.5 µL (LAND, Hongkong), cel-miR-39 reverse transcription primer 0.5 µL (LAND, Hongkong), 5× buffer 5 µL, M-MLV Reverse Transcriptase 1 µL (Promega); 42°C for 60 min, and 85°C for 10 min. The cDNA obtained by reverse transcription was diluted (1∶20) for real-time PCR. The reaction system used was as follows: miRNA specific forward primer 0.5 µL (Invitrogen), miRNA general primer 0.5 µL (Invitrogen), 2× SYBR Green PCR Master Mix 10 µL (Toyobo, Japan), cDNA 5 µL; DEPC-treated water was added to give a final reaction volume of 20 µL. The real-time PCR reaction conditions were: 95°C for 5 min, followed by 40 cycles of 95°C for 15 sec, 62°C for 15 sec, and 72°C for 32 sec. After adding TRIzol, the samples were supplemented with cel-miR-39 as an endogenous control (GenePharma, China), as described previously [12] (Primer sequences are shown in Table 1).

### Population Study

22 subjects were divided into 2 groups: LVH group (11 patients with LVH) and control group (11 subjects without LVH) (Table 2). Inclusion criteria for the LVH group included diagnosis by echocardiography: interventricular septal thickness at end-diastole (IVSd) and/or left ventricular posterior wall thickness at end-diastole (LVPWd) ≥1.2 cm; ejection fraction (EF) >40%. Patients with systolic heart failure, tumors, infection (acute phase), brain natriuretic peptide (BNP) >400 ng/L, Parkinson’s disease, idiopathic pulmonary fibrosis, shock or cirrhosis were excluded, as were patients treated recently with interferon or phenobarbitone. Whole blood was collected from the patients (fasted for 8 h), into a tube containing EDTA. The Institutional Ethics Committee of the Second Hospital Affiliated to Guangzhou Medical University approved the study, and all patients gave a written informed consent.

**Table 2:** clinical characteristics of patients with and without LVH.

	Control(n = 11)	LVH(n = 11)	*P*
Age(years)	69.0±12.2	73.9±12.4	0.362
Male/female(n/n)	4/7	6/5	0.670
Smoking,n	1(9.1%)	5(45.5%)	0.149
DM, n(%)	5(45.5%)	2(18.2%)	0.361
Hypertension,n(%)	8(72.7%)	11(100.0%)	0.214
ACS,n(%)	4(36.4%)	4(36.4%)	1.000
Af,n(%)	1(9.1%)	1(9.1%)	1.000
Fasting glucse(mmol/L)	6.23±2.14	5.07±0.83	0.110
SBP(mmHg)	136.3±25.3	158.3±30.2	0.079
DBP(mmHg)	74.5±14.1	85.5±14.9	0.091
TC(mmol/L)	5.04±1.23	5.00±1.11	0.927
TG(mmol/L)	1.39±0.57	1.16±0.48	0.314
HDL(mmol/L)	1.12±0.27	1.26±0.38	0.320
LDL(mmol/L)	2.93±0.91	2.64±0.68	0.417
Cr(µmol/L)	129.6±120.5	105.1±72.4	0.949
CK-MB(U/L)	28.5±44.3	15.8±10.8	0.331
BNP(ng/L)	169.9±112.0	130.4±112.2	0.370
EF(%)	64.6±6.2	62.9±6.7	0.546
LV(cm)	4.418±0.506	4.318±0.536	0.658
IVSd(cm)	0.991±0.094	1.256±0.098	0.000
LVPWd(cm)	0.946±0.104	1.256±0.088	0.000

DM = diabetes mellitus, ACS = acute coronary syndrome, Af = atrial fibrillation, SBP = systolic blood pressure, DBP = diastolic blood pressure, TC = total cholesterol, TG = total glyceride, HDL = high-density lipoprotein, LDL = low-density lipoprotein, Cr = creatinine, CK-MB = creatine kinase-MB, LV = left ventricular diameter.

### Statistical Analysis

All results for continuous variables were expressed as mean ± SEM, unless otherwise indicated. The Shapiro-Wilk test was used to establish whether the continuous data followed a normal distribution. For data that were normally distributed, Levene’s test for homogeneity of variance was then performed. For group-wise comparisons, a Student’s *t* test, Mann-Whitney U Test or ANOVA were used, as appropriate. For categorical variables, a Fisher’s exact test was employed. Pearson correlation was used to measure the strength of the association between microRNA level and ventricular wall thickness. All tests were performed as 2-sided tests, and a significance level of *P*<0.05 was considered to indicate statistical significance. Statistical analyses were performed using PASW Statistics 18.0 software.

## Results

### Up-regulation of Autophagy and Down-regulation of miR-30 in Rats with TAAC-Induced Cardiac Hypertrophy

**Figure 1:**
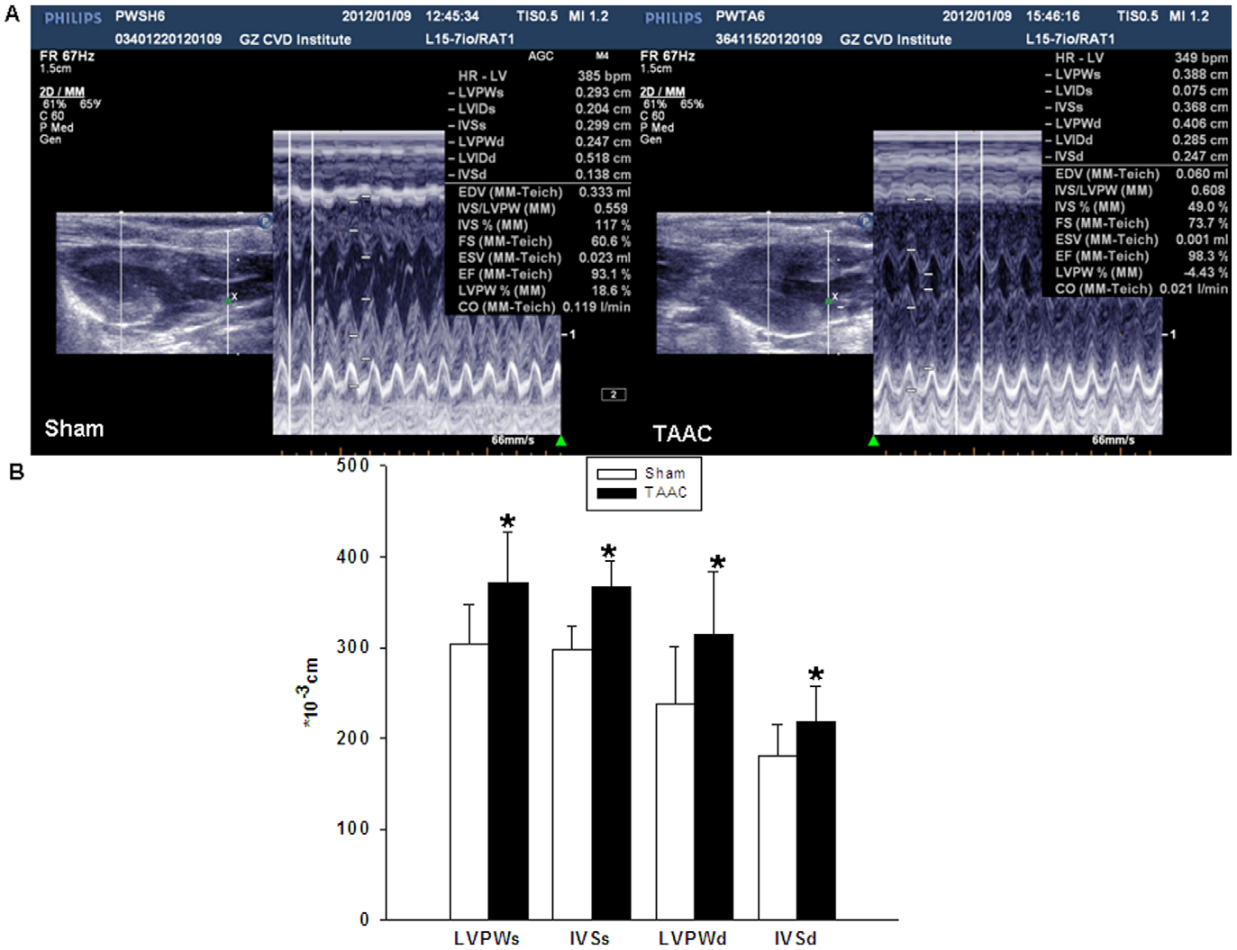
Ventricular wall thickness was assessed using echocardiography in Wistar rats after TAAC surgery. (**A**) Representative ultrasound through left ventricular from rats after Sham operation and transverse abdominal aortic constriction (TAAC)for 4 weeks. (**B**) Quantitative analysis of echocardiography for the left ventricular posterior wall thickness at end-systole (LVPWs), interventricular septal thickness at end-systole (IVSs), left ventricular posterior wall thickness at end-diastole (LVPWd) and interventricular septal thickness at end-diastole (IVSd), and comparison was performed between TAAC (n = 8) and Sham group(n = 6). Data are presented as means ± SEM. *P<0.05 compared with Sham.

**Figure 2:**
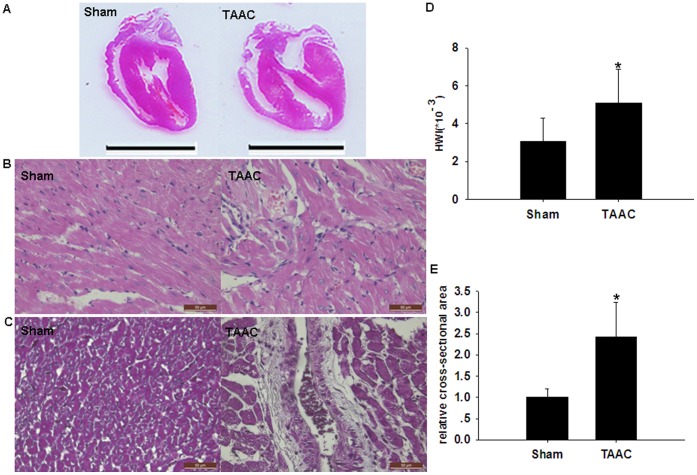
Pathological examination of hearts from Wistar rats after TAAC surgery. (**A**) Scanning images of the HE-stained paraffin sections of the hearts from Sham and TAAC rats, and the scale bar: 1 cm. (**B**) Microscopic images of the HE-stained paraffin sections of the hearts from Sham and TAAC rats, and the scale bar: 50 µm. Cardiac tissue color was lighter with inhomogeneous staining, numerous nuclear-free regions were evident, and an increased nucleolar density in muscle fibers in regions of atrophy was observed. (**C**) Microscopic images of the Masson’s-stained paraffin sections of the hearts from Sham and TAAC rats, and the scale bar: 50 µm. The regions of fibroplasia around blood vessels were presented as blue. (**D**)Heart weight index (HWI) was assessed by heart weight-to-body weight ratio. (**E**) The relative cross-sectional area was evaluated by calculating the ratio of the cardiac muscle fiber surface area of the microscopic images of the paraffin sections. Data are presented as means ± SEM. *P<0.05 compared with Sham.

Echocardiography showed that the cardiac LVPW and IVS of rats in the TAAC group were significantly higher than those in the Sham group (Figure 1). After echocardiography, the rats were sacrificed and the hearts harvested. The heart volume, heart weight index (HWI, which was assessed by heart weight-to-body weight ratio) and the ventricular wall thickness were significantly greater in rats from the TAAC group than in those from the Sham group; in addition, the papillary muscles and trabeculae carneae cordis were more coarse in appearance in the TAAC group than in the Sham group. Microscopic examination of the tissue, after staining, revealed characteristic features of myocardial hypertrophy in the TAAC group. In samples with HE staining, regions of myocardial hypertrophy were lighter in color with inhomogeneous staining, and showed numerous nuclear-free regions, with an increased nucleolar density in regions of muscle fiber atrophy. In samples with Masson staining, areas of fibroplasia were observed under the endomembrane, with an increased thickness of vessel walls, fibroplasia around blood vessels, and an increased cardiac muscle fiber surface area (Figure 2). These results indicated that the rat model of cardiac hypertrophy had been successfully established.

**Figure 3:**
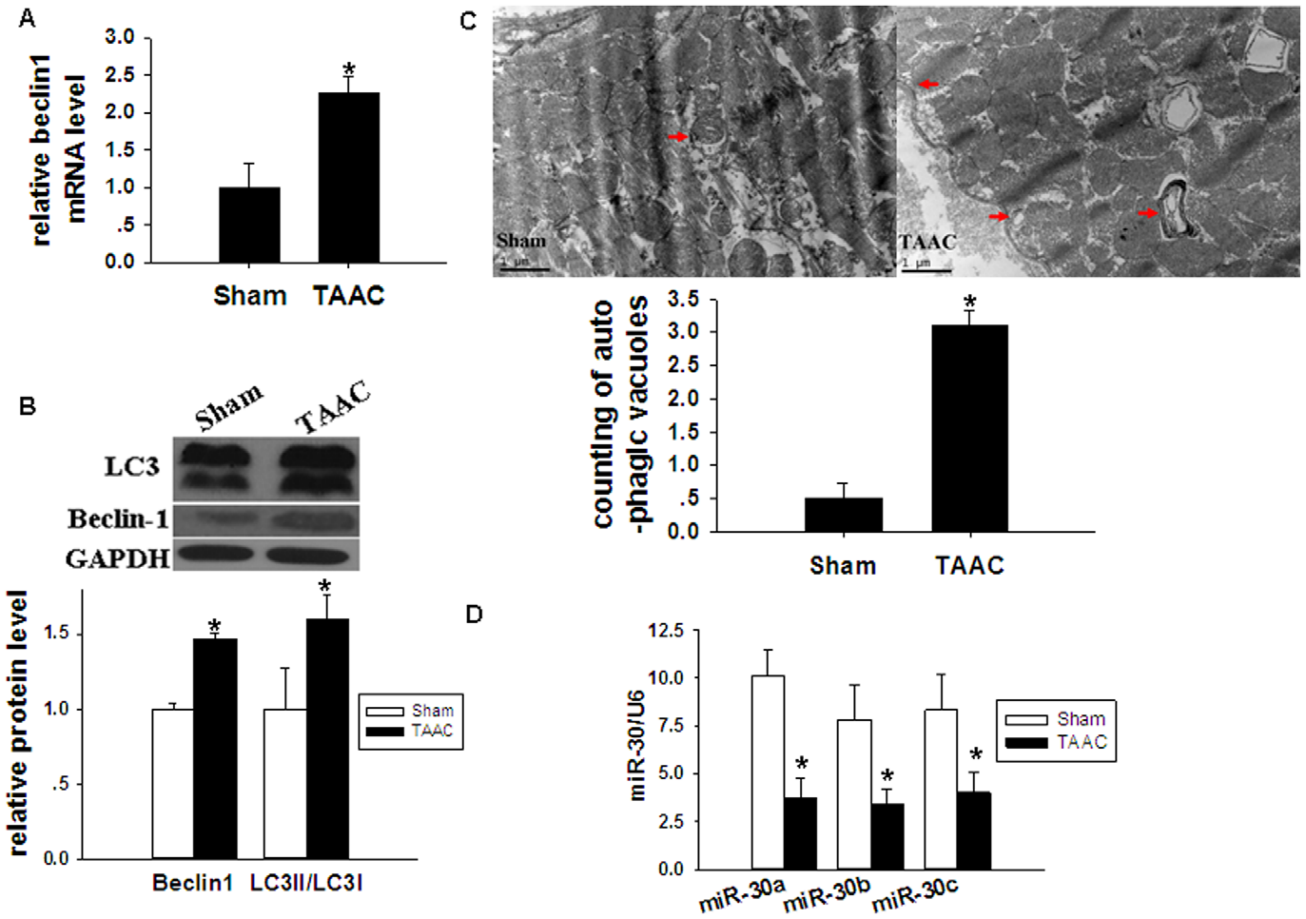
Autophagy and miR-30 expression in rats after TAAC surgery. The relative expression of mRNA level, microRNA level, protein, and autophagic vacuoles in left ventricular tissues from Sham and TAAC rats was analyzed 4 weeks after the operation. (**A**) Relative mRNA expression level of *beclin-1* was analyzed by qRT-PCR. 18S was used as an internal control. (**B**) Relative expression of autophagy-related proteins, beclin-1 and LC3, was analyzed by Western blotting. GAPDH was used as an internal control. (**C**) Autophagic vacuoles (arrows indicated) were detected by transmission electron microscopy, at a magnification of ×13500. Scale bar: 1 µm. (**D**) Relative miR-30a, miR-30b and miR-30c expression which was presented as 2^−Δct^*10^−3^ was analyzed by qRT-PCR. U6 was used as an internal control. Data are presented as means ± SEM. *P<0.05 compared with Sham.

The expression of *beclin-1* mRNA and beclin-1 protein significantly increased in rats from the TAAC group, compared with those of the Sham group. Expression of autophagy-related protein LC3II/LC3I (Figure 3A and 3B), and the number of autophagic vacuoles in the heart, were also significantly higher in the TAAC group (Figure 3C), compared with the Sham group. The expression of miR-30a, miR-30b and miR-30c was significantly lower in rats from the TAAC group, compared with those in the Sham group (Figure 3D).

### Myocardial Hypertrophy Induced by Excessive Activation of Autophagy in Cardiomyocytes

**Figure 4:**
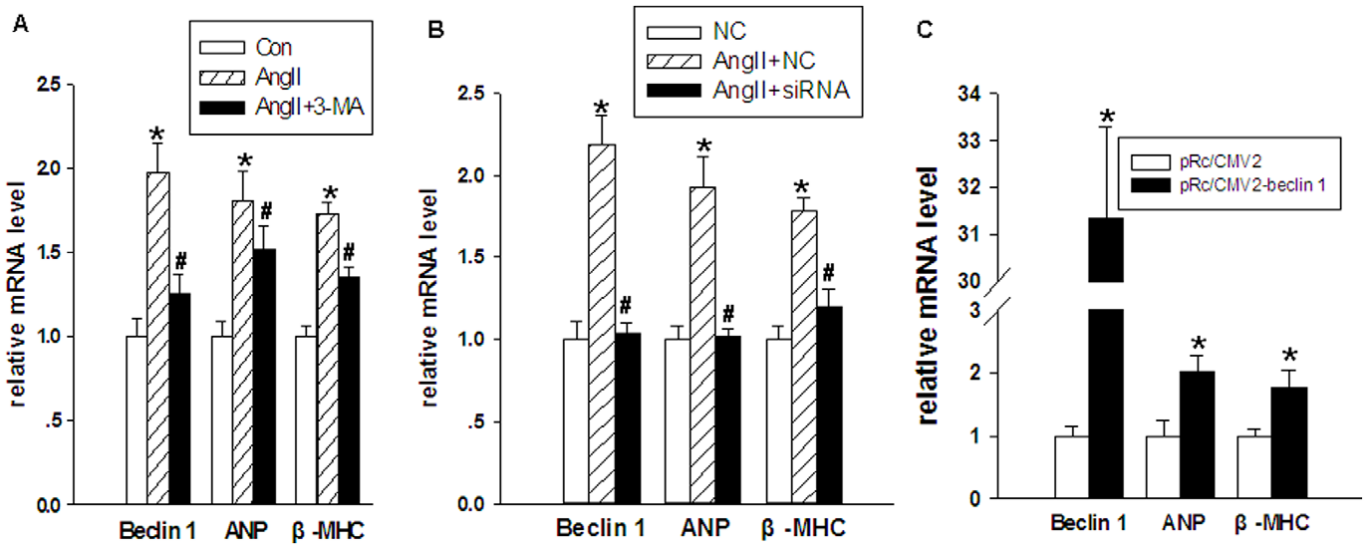
The role of the *beclin-1* gene in the development of myocardial hypertrophy. The relative mRNA level of *beclin-1*, *ANP*, and *β-MHC* in cardiomyocytes was analyzed by qRT-PCR. 18S was used as an internal control. Data are presented as means ± SEM. (**A**) Evaluation of the influence of 3-methyladenine (3-MA) on the expression of *beclin-1* and hypertrophy related genes. Con: control group, Ang II: treated with 1 µmol/L Angiotensin II, and Ang II+3-MA: treated with 1 µmol/L Angiotensin II and 10 mmol/L 3-MA. *P<0.05 compared with Con; # *P*<0.05 compared with Ang II group. (**B**) Evaluation of the influence of *beclin-1* specific siRNA on the expression of *beclin-1* and hypertrophy related genes. NC: treated with lentivirus containing negative control of *beclin-1*-specific siRNA, Ang II+ NC: treated with lentivirus containing negative control of *beclin-1*-specific siRNA and 1 µmol/L Angiotensin II, and Ang II+siRNA: treated with lentivirus containing *beclin-1*-specific siRNA and 1 µmol/L Angiotensin II. *P<0.05 compared with NC group; # *P*<0.05 compared with Ang II+NC group. (**C**) Evaluation of the influence of pRc/CMV2-beclin-1 vector on the expression of *beclin-1* and hypertrophy related genes. pRc/CMV2: transfected with pRc/CMV2 vector, and pRc/CMV2-beclin-1: transfected with pRc/CMV2-beclin-1. *P<0.05 compared with pRc/CMV2 group.

Compared with the controls, the expression of *beclin-1*, *ANP* (atrial natriuretic peptide) and *β-MHC* (beta-myosin heavy chain) genes was up-regulated in neonatal cardiomyocytes stimulated with Ang II. Compared with the Ang II-treated neonatal cardiomyocytes, the expression of *beclin-1*, *ANP* and *β-MHC* genes was down-regulated in cardiomyocytes treated with either 3-MA+Ang II, or *beclin-1-*specific siRNA+Ang II (Figure 4A and 4B). Hence, hypertrophy-related gene expression in these cardiomyocytes was attenuated by inhibition of excessive autophagy. The expression of myocardial hypertrophy-related genes increased by over-expression of the *beclin-1* gene, as compared with cardiomyocytes transfected with the pRc/CMV2 vector (Figure 4C).

**Figure 5:**
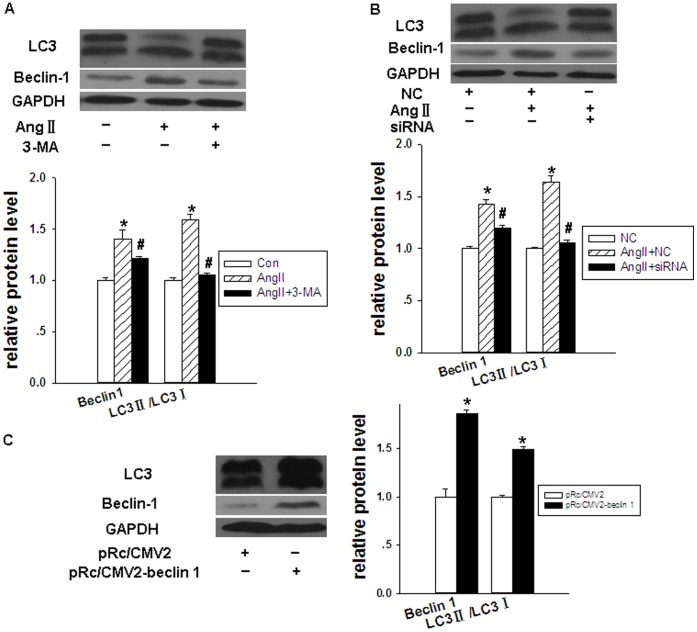
Expression of autophagy-related protein in cardiomyocytes varies with that of the *beclin-1* gene. The relative expression of autophagy-related protein in cardiomyocytes was analyzed by Western blotting. GAPDH was used as an internal control. Data are presented as means ± SEM. (**A**) Evaluation of the influence of 3-MA on the expression of LC3II/LC3I and beclin-1 proteins. Con: control group, Ang II: treated with 1 µmol/L Angiotensin II, and Ang II+3-MA: treated with 1 µmol/L Angiotensin II and 10 mmol/L 3-MA. *P<0.05 compared with Con group; # *P*<0.05 compared with Ang II group. (**B**) Evaluation of the influence of *beclin-1* specific siRNA on the expression of autophagy-related proteins. NC: treated with lentivirus containing negative control of *beclin-1*-specific siRNA, Ang II+ NC: treated with lentivirus containing negative control of *beclin-1*-specific siRNA and 1 µmol/L Angiotensin II, and Ang II+siRNA: treated with lentivirus containing *beclin-1*-specific siRNA and 1 µmol/L Angiotensin II. *P<0.05 compared with NC group; # *P*<0.05 compared with Ang II+NC group. (**C**) Evaluation of the influence of pRc/CMV2-beclin-1 vector on LC3II/LC3I and beclin-1 proteins. pRc/CMV2: transfected with pRc/CMV2 vector, and pRc/CMV2-beclin-1: transfected with pRc/CMV2-beclin-1. *P<0.05 compared with pRc/CMV2 group.

**Figure 6:**
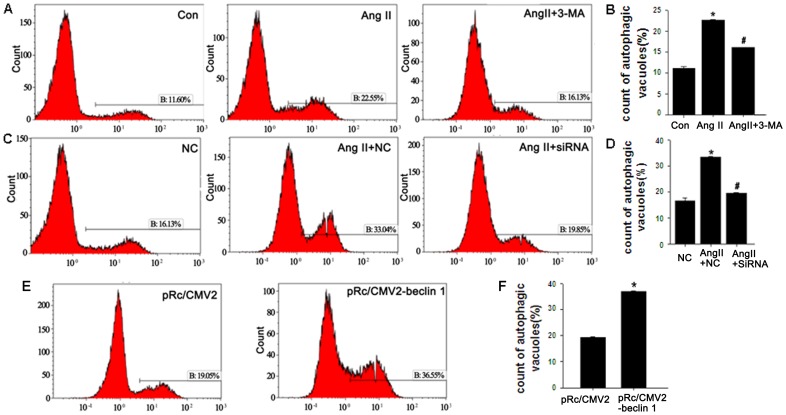
The percentage of autophagic vacuoles varies with *beclin-1* gene expression. The autophagic vacuoles was analyzed by calculating MDC-stained cardiomyocytes using flow cytometry. (**A, C** and **E**) Representative percentage of autophagic vacuoles measured in MDC-stained cardiomyocytes using flow cytometry. (**B**) Evaluation of the influence of 3-MA on autophagic vacuoles. Con: control group, Ang II: treated with 1 µmol/L Angiotensin II, and Ang II+3-MA: treated with 1 µmol/L Angiotensin II and 10 mmol/L 3-MA. *P<0.05 compared with Con group; # *P*<0.05 compared with Ang II group. (**D**) Evaluation of the influence of *beclin-1* specific siRNA on autophagic vacuoles. NC: treated with lentivirus containing negative control of *beclin-1*-specific siRNA, Ang II+ NC: treated with lentivirus containing negative control of *beclin-1*-specific siRNA and 1 µmol/L Angiotensin II, and Ang II+siRNA: treated with lentivirus containing *beclin-1*-specific siRNA and 1 µmol/L Angiotensin II. *P<0.05 compared with NC group; # *P*<0.05 compared with Ang II+NC group. (**F**) Evaluation of the influence of pRc/CMV2-beclin-1 vector on autophagic vacuoles. pRc/CMV2: transfected with pRc/CMV2 vector, and pRc/CMV2-beclin-1: transfected with pRc/CMV2-beclin-1. *P<0.05 compared with pRc/CMV2 group. Data are presented as means ± SEM.

**Figure 7:**
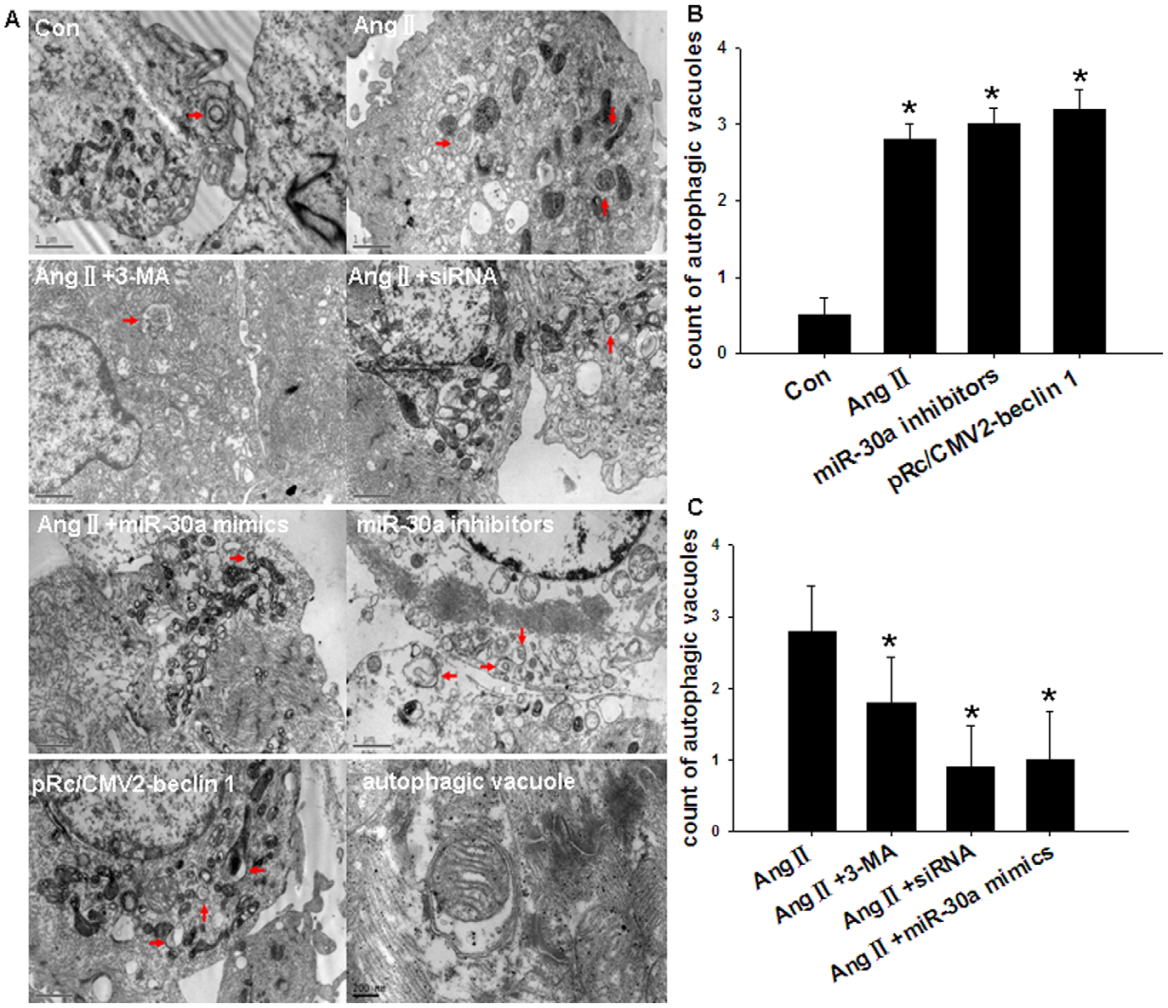
Autophagic vacuole number varies with *beclin-1* gene expression. The autophagic vacuoles was analyzed by transmission electron microscopy. (**A**) Representative autophagic vacuoles (arrows indicated) were detected by transmission electron microscopy. Magnification of the first seven images: ×13500. The final image showed an autophagic vacuole under x46000 magnificationor: part of the cytoplasm and the organelle were packaged into specific double-membrane structures. Scale bar of the first seven images: 1 µm. Scale bar of the final image:200 nm.1 µm. –regulation. (**B**) Evaluation of the influence of up-regulation of *beclin-1* ****on autophagic vacuoles. Con: control group, Ang II: treated with 1 µmol/L Angiotensin II, miR-30a inhibitors: treated with miR-30a inhibitors, and pRc/CMV2-beclin-1: treated with pRc/CMV2-beclin-1. *P<0.05 compared with Con group. (**C**) Evaluation of the influence of down-regulation of *beclin-1* ****on autophagic vacuoles. Ang II: treated with 1 µmol/L Angiotensin II, Ang II+3-MA: treated with 1 µmol/L Angiotensin II and 10 mmol/L 3-MA, Ang II+siRNA: treated with *beclin-1*-specific siRNA and 1 µmol/L Angiotensin II, and Ang II+miR-30a mimics: treated with 1 µmol/L Angiotensin II and miR-30a mimics. *P<0.05 compared with Ang II group. Data are presented as means ± SEM.

Inhibition or over-expression of the *beclin-1* gene in cardiomyocytes was associated with inhibition or up-regulation of autophagy, respectively. Over-expression of the *beclin-1* gene was induced by Ang II or by transfection of a *beclin-1* gene expression vector. After over-expression of the *beclin-1* gene in cardiomyocytes, the expression of LC3II/LC3I and beclin-1 proteins significantly increased (Figure 5). The inhibition of *beclin-1* gene expression was achieved using 3-MA or *beclin-1-*specific siRNA. After down-regulation of *beclin-1* gene expression in cardiomyocytes, the expression of LC3II/LC3I and beclin-1 proteins significantly decreased (Figure 5A and 5B). The percentage and number of autophagic vacuoles were measured using flow cytometry of MDC-stained cardiomyocytes (Figure 6) or electron microscopy (Figure 7). The results of these experiments confirmed that alterations in cardiomyocyte *beclin-1* gene expression can cause corresponding changes in autophagy.

### miR-30-regulated Autophagy in Cardiomyocytes

**Figure 8:**
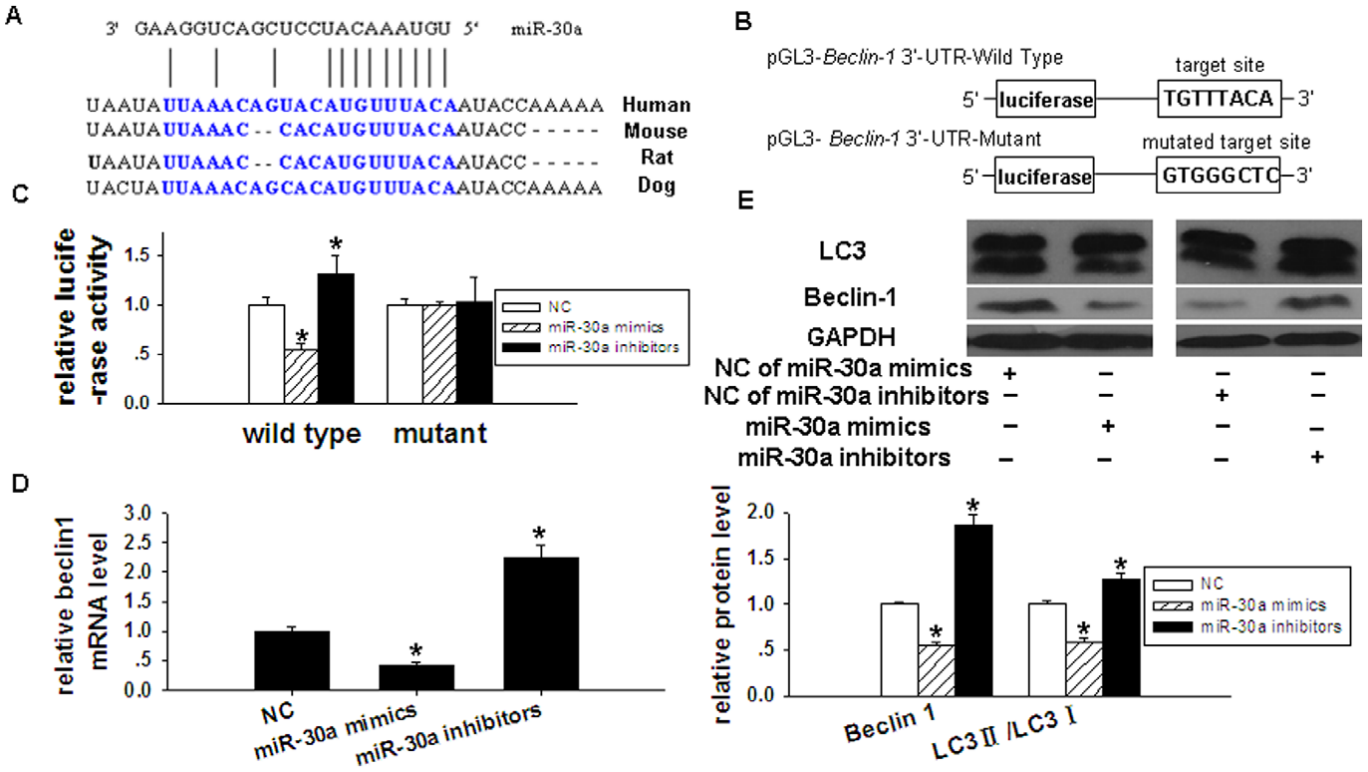
*Beclin-1* is a target gene of miR-30a. (**A**) Sequence alignment between miR-30a and the 3′-UTR of *beclin*-*1* in several species. (**B**) The schematic diagram of the plasmid of pGL3-*Beclin-1* 3′-UTR-Wild Type and pGL3- *Beclin-1* 3′-UTR-Mutant. The wild-type and mutated sequences of the target site of miR-30a on the 3′-UTR of *Beclin-1* were shown. (**C**) Analysis of the relative luciferase activity in cardiomyocytes cotransfected with pGL3-*Beclin-1* 3′-UTR-Wild Type or pGL3-*Beclin-1* 3′-UTR-Mutant and miR-30a mimics, miR-30a inhibitors and negative control.*P<0.05 compared with NC group. (**D**) Evaluation of the influence of miR-30a on mRNA level of *beclin-1* in cardiomyocytes. *P<0.05 compared with NC group. (**E**) Evaluation of the influence of miR-30a on expression of beclin-1 and LC3II/LC3I proteins in cardiomyocytes. *P<0.05 compared with NC group.

Bioinformatic predictions suggested that the 3′UTR of the *beclin-1 *gene would have one binding site for miR-30a, and that the *beclin-1 *gene may be a target gene for miR-30a (Figure 8A). In cardiomyocytes transfected with pGL3-*Beclin-1* 3′-UTR-Wild Type, transduction with an miR-30a mimic decreased the relative luciferase activity by 45.4%, relative to cells treated with a negative control. In cardiomyocytes transfected with pGL3-*Beclin-1* 3′-UTR-Wild Type, transduction with an miR-30a inhibitor increased the relative luciferase activity 1.32-fold, compared with cells treated with a negative control. In cardiomyocytes transfected with pGL3-*Beclin-1* 3′-UTR-Mutant, there were no significant differences in the relative luciferase activities between cells treated with an miR-30a mimic or inhibitor and cells treated with a negative control (Figure 8C). In summary, these findings suggest that *beclin-1* was a target gene of miR-30a. To determine the effect of miR-30a on the expression of *beclin-1*, the mRNA and protein expression levels of *beclin-1* were measured in cardiomyocytes treated with either an miR-30a mimic or an miR-30a inhibitor. Compared with the negative control, the expression of *beclin-1 *mRNA decreased by 57.5% in cells treated with an miR-30a mimic, and increased by 2.25-fold in cells treated with an miR-30a inhibitor (Figure 8D). Compared with the negative control, beclin-1 protein levels decreased by 44.7% in cells treated with an miR-30a mimic, and increased by 1.87-fold in cells treated with an miR-30a inhibitor (Figure 8E).

**Figure 9:**
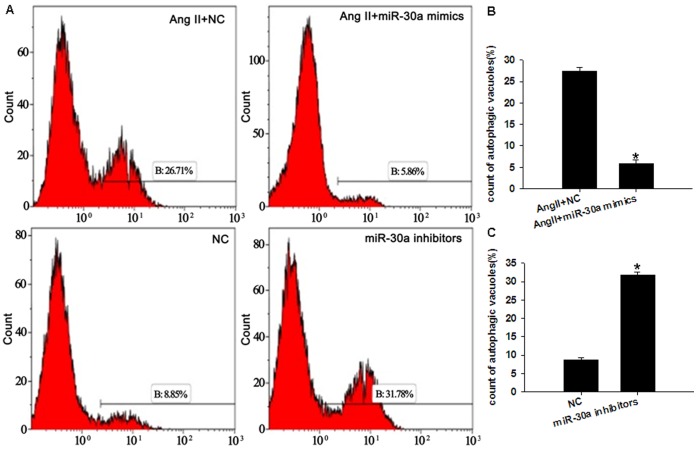
MiR-30a regulates the percentage of autophagic vacuoles. The autophagic vacuoles was analyzed by calculating MDC-stained cardiomyocytes using flow cytometry. (**A**)Representative percentage of autophagic vacuoles measured in MDC-stained cardiomyocytes using flow cytometry. (**B**) Evaluation of the influence of miR-30a mimics on autophagic vacuoles. *P<0.05 compared with Ang II +NC group. (**C**) Evaluation of the influence of miR-30a inhibitors on autophagic vacuoles. *P<0.05 compared with NC group.

miR-30a was found to not only induce changes in beclin-1 protein expression, but also alter the expression of other autophagy-related proteins. Compared to cardiomyocytes treated with a negative control, the expression of LC3II/LC3I protein decreased by 41.3% in cells treated with an miR-30a mimic, and increased 1.28-fold in cells treated with an miR-30a inhibitor (Figure 8E). The number of autophagic vacuoles in the visual field (×13500) significantly increased in cells treated with an miR-30a inhibitor, compared with control cells (0.5±0.224 vs 3.0±0.221, *P = *0.000). Compared with Ang II-stimulated cells, the number of autophagic vacuoles decreased in cardiomyocytes treated with Ang II+miR-30a mimic (2.8±0.632 vs 1.0±0.667, *P = *0.000) (Figure 7). Autophagic vacuole percentages were measured in MDC-stained cardiomyocytes using flow cytometry: these data confirmed that miR-30 functioned to regulate autophagy in cardiomyocytes (Figure 9).

### Downre-gulation of miR-30 Leads to Myocardial Hypertrophy

**Figure 10:**
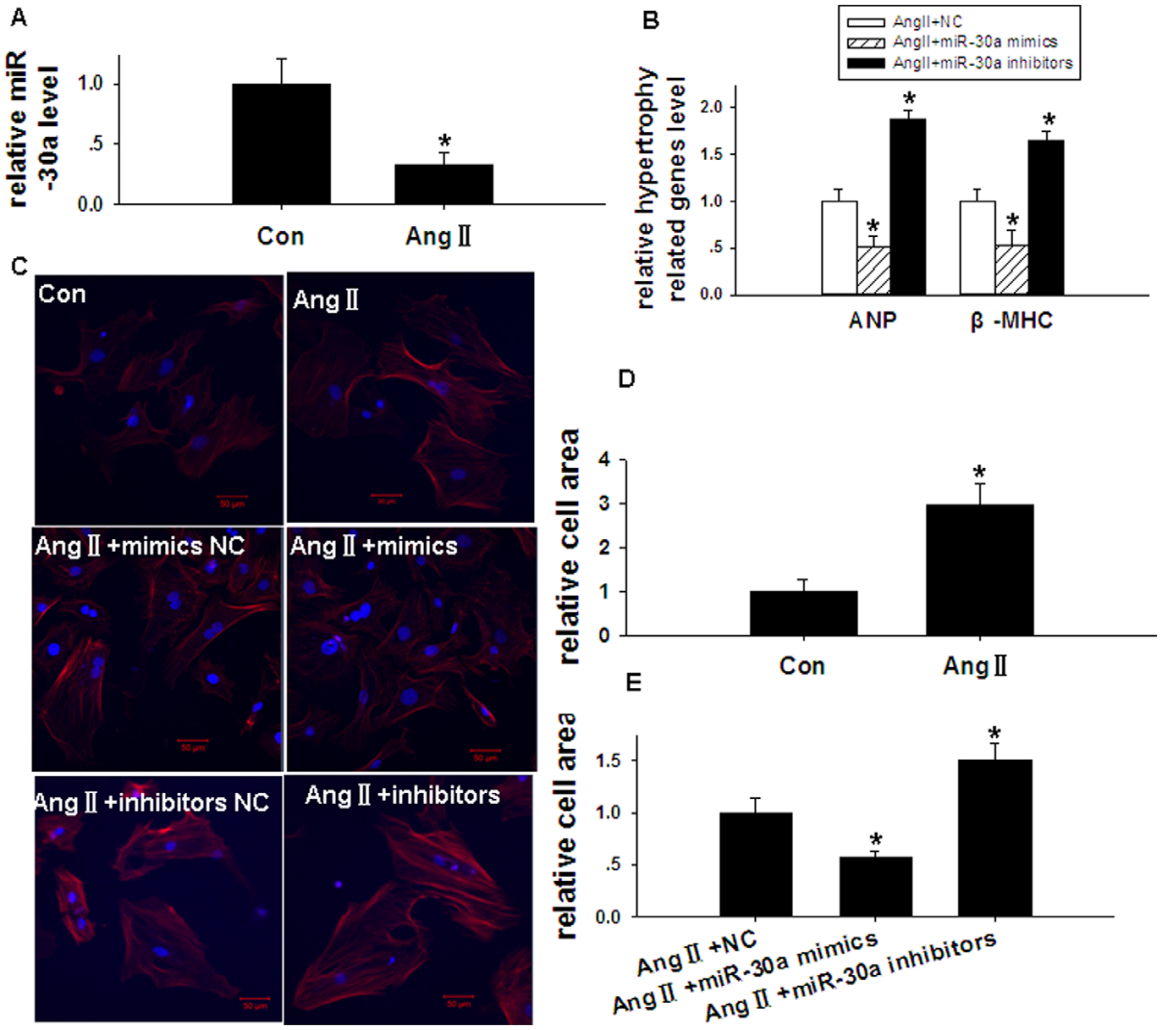
Down-regulation of miR-30 leads to myocardial hypertrophy. (**A**) Relative expression of miR-30a in Ang II-stimulated cardiomyocytes (treated with 1 µmol/L Angiotensin II) and that in unstimulated cells by real-time RT-PCR relative to U6. *P<0.05 compared with Con group. (**B**) Evaluation of the influence of miR-30a on mRNA level of *ANP* and *β-MHC* in hypertrophic cardiomyocytes. *P<0.05 compared with Ang II+NC group. (**C**) Morphological changes were observed using confocal microscopy in cardiomyocytes stained with Alexa Fluor®555 Phalloidin and DAPI. Summarized data are shown in (**D** and **E**). (**D**) Evaluation of the influence of Ang II on relative cell area (the cardiac muscle fiber surface area ratio) in cardiomyocytes. *P<0.05 compared with Con group. (**E**) Evaluation of the influence of miR-30a on relative cell area in hypertrophic cardiomyocytes. *P<0.05 compared with Ang II+NC group.

The expression of miR-30a in Ang II-stimulated cardiomyocytes was only 32.9% of that in unstimulated cells (Figure 10A). To study the relationship between the down-regulation of miR-30a and the development of myocardial hypertrophy, the expression of *ANP* and *β-MHC* was measured. In hypertrophic cardiomyocytes (hypertrophy induced by Ang II), treatment with an miR-30a mimic decreased the expression of *ANP* and *β-MHC* by 48.3% and 46.5%, respectively, relative to the negative control. Conversely, in hypertrophic cardiomyocytes (induced by Ang II), treatment with an miR-30a inhibitor increased the expression of *ANP* and *β-MHC* by 1.88- and 1.64-fold, respectively, relative to the negative control (Figure 10B). Morphological changes in these cardiomyocytes were observed using confocal microscopy (Figure 10C). The morphological observations indicated that the surface area of hypertrophic cardiomyocytes (induced by Ang II) was 2.95-fold that of untreated cells (Figure 10D). Furthermore, compared with hypertrophic cardiomyocytes treated with Ang II+negative control, the cell surface area decreased by 42.2% in cardiomyocytes treated with Ang II+miR-30a mimic, and increased 1.50-fold in cardiomyocytes treated with Ang II+miR-30a inhibitor (Figure 10E).

### Circulating Levels of miR-30 Increased in Rats Following TAAC Surgery, and in Patients with LVH

**Figure 11:**
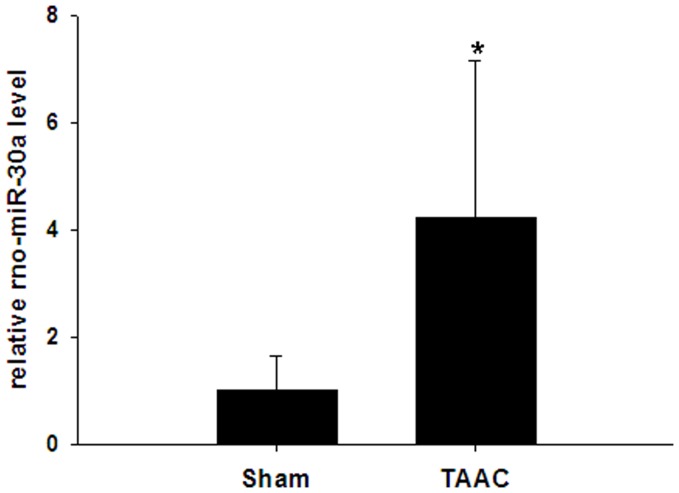
circulating miR-30 expression in rats from the TAAC group. The circulating miR-30a in rats (miR-30a in Rattus norvegicus, rno-miR-30a) from TAAC group and Sham group was analyzed by real-time RT-PCR 4 weeks after the operation. cel-miR-39 was used an endogenous control. *P<0.05 compared with Sham group.

**Figure 12:**
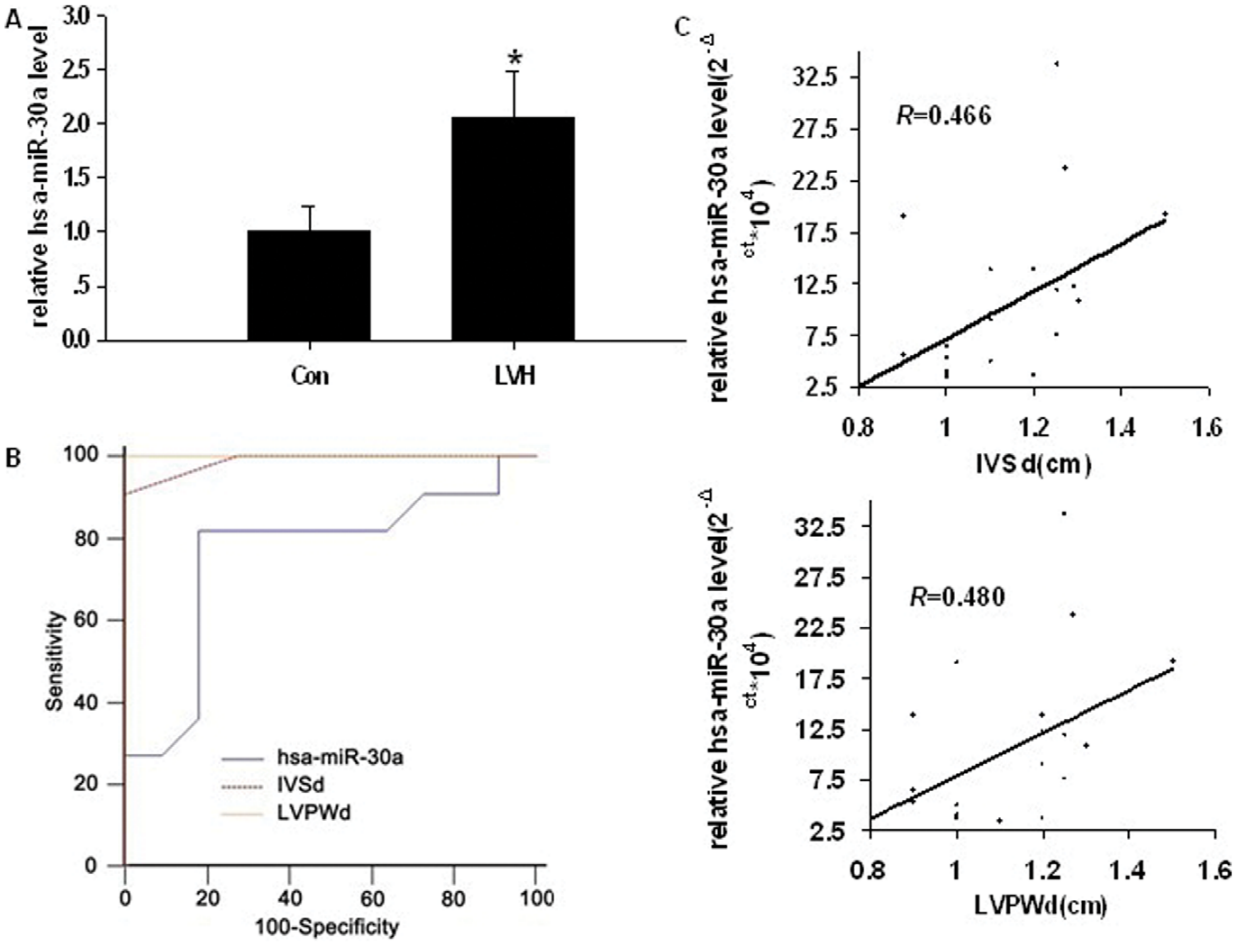
Relationship between the circulating miR-30 level and ventricular wall thickness. 22 subjects were divided into two groups: LVH group (n = 11) and control group (n = 11). (**A**) Comparison of the circulating miR-30a (miR-30a in Homo sapiens, has-miR-30a) between patients with LVH and patients without LVH by real-time RT-PCR relative to cel-miR-39. *P<0.05 compared with Con group. (**B**) Evaluation of the sensitivity and specificity of has-miR-30a on the diagnosis of LVH was performed by analyzing the ROC curve using MedCalc11.3 software. (**C**) The association between circulating has-miR-30a level and ventricular wall thickness was assessed by Pearson correlation analysis. *R*: Pearson correlation coefficient.

The concentration of miR-30a in the plasma of rats from the TAAC group was 4.23-fold greater than that of rats from the Sham group (*P = *0.000, Figure 11). With the exception of IVSd and LVPWd, there were no significant differences in the clinical characteristics between patients with LVH and those without LVH (Table 1). Since 11 LVH patients suffered from primary hypertension, their LVH may be associated with abnormal blood pressure. The level of miR-30a expression was 2.07-fold higher in patients with LVH, compared with patients without LVH (*P = *0.040, Figure 12A). A receiver operating characteristic (ROC) curve showed that the areas under the curve (AUC) for the plasma level of miR-30a, for IVSd and for LVPWd were 0.760, 0.988 and 1.000, respectively. Use of plasma miR-30 levels for the diagnosis of LVH reached statistical significance (*P = *0.039, Figure 12B). Pearson correlation analysis indicted that the level of miR-30a expression in plasma was positively associated with IVSd and LVPWd (Figure 12C) (*R* = 0.466 and 0.480, *P = *0.029 and 0.024, respectively,).

## Discussion

Autophagy was originally defined as the process of sequestration of intracellular components and their subsequent degradation by lysosomal vacuoles [13]. This process is likely the main mechanism involved in the degradation of long-lived proteins and cytoplasmic organelles. In our study, we found that the expression of the *beclin-1* gene and autophagy in rat heart were significantly increased in the TAAC group relative to the Sham group. Both autophagy and the expression of the *beclin-1* gene increased in Ang II-stimulated neonatal cardiomyocytes, compared with control cells. Moreover, inhibition of the *beclin-1* gene in cardiomyocytes was associated with reduced autophagic activity, whereas over-expression of *beclin-1* resulted in enhanced autophagy. We were interested in determining whether over-expression of the *beclin-1* gene and excessive autophagy are mechanisms that mediate Ang II-induced myocardial hypertrophy. We found that silencing of the *beclin-1* gene by RNA interference caused decreased expression of genes related to myocardial hypertrophy, whereas over-expression of the *beclin-1* gene resulted in an enhancement of the expression of genes related to myocardial hypertrophy. Based on these observations, we propose that *beclin-1* gene over-expression and excessive autophagy mediate Ang II-induced myocardial hypertrophy. When autophagy is excessively up-regulated in cardiomyocytes, redundant cytoplasmic organelles and spectrin are cleared by this autophagic process. Clearance of redundant cytoplasmic organelles, such as mitochondria, leads to disturbances in normal physiological function, while protein degradation via excessive autophagy results in a reactive acceleration of protein synthesis and the development of myocardial hypertrophy. Therefore, we speculate the existence of the following autophagy-associated pathway:Ang II ↑→*beclin-1* gene expression ↑→ excessive autophagy → myocardial hypertrophy.

MicroRNAs are endogenous (≈22-nucleotide) non-coding RNA molecules that regulate gene expression at the post-transcriptional level, by pairing with partially complementary sites in the 3′ UTR of the targeted mRNA, leading either to degradation or to translational repression. MicroRNAs control development, and are critically involved in many biological processes in health and disease, including cardiovascular diseases [14], [15]. Bioinformatics software predicts that miR-30a may regulate the expression of *beclin-1.* The function of the miR-30 family is similar to that of other microRNAs. The miR-30 family is associated with the development of tumors and other diseases (of the nervous, genital, circulatory, alimentary, and respiratory systems), as well as adipogenesis, cellular senescence, drug metabolism and cell differentiation [16]–[31]. A study by Duisters et al. [10] found that miR-30 family members were significantly down-regulated in mouse hypertrophic hearts and in cardiac biopsies from patients with LVH. The pro-fibrotic protein, connective tissue growth factor (CTGF), was down-regulated by miR-30c, such that structural changes in the extracellular matrix of the myocardium were controlled by miR-30c. In another study, chronic alcohol intake in mice has been reported to lead to cardiac hypertrophy and down-regulation of miR-30a [32].

Our experiments revealed that expression of the miR-30 family was down-regulated in cardiomyocytes of rats from the TAAC group, and that miR-30a expression decreased in hypertrophic cardiomyocytes that had been treated with Ang II. It was thus of interest to investigate whether the down-regulation of miR-30 might mediate Ang II-induced myocardial hypertrophy. After treatment of cardiomyocytes with miR-30a mimic, the expression of genes related to myocardial hypertrophy decreased, and the hypertrophic cardiomyocytes showed improvements in their morphology. Furthermore, transduction of an miR-30a inhibitor into cardiomyocytes, to impair the function of miR-30a, caused an up-regulation of the expression of genes related to myocardial hypertrophy, with more severe morphological changes characteristic of hypertrophy. Therefore, we postulate that down-regulation of miR-30a expression mediates Ang II-induced myocardial hypertrophy, as follows:Ang II ↑→miR-30a ↓→ myocardial hypertrophy.

In additional experiments, we further demonstrated that *beclin-1* is the target gene of miR30a, and that miR-30a binds with the 3′UTR of *beclin-1*. The expression of *beclin-1* was down-regulated in cardiomyocytes treated with an miR-30a mimic, and up-regulated in those treated with an miR-30a inhibitor. The results of our study also indicate that activation of autophagy was enhanced in Ang II-induced hypertrophic cardiomyocytes that had been treated with an miR-30a inhibitor, wherease autophagy was inhibited in Ang II-treated cells by over-expression of an miR-30a mimic. Consistent with our observations, the study of Zhu et al. suggested that cardiac autophagy may be a maladaptive response to hemodynamic stress [5]. Taken together, these results provide strong evidence that autophagy mediates the development of myocardial hypertrophy in cardiomyocytes: a down-regulation of miR-30 induced by Ang II leads to excessive autophagy in cardiomyocytes, thereby promoting myocardial hypertrophy. A possible pathway by which Ang II may cause myocardial hypertrophy is as follows: Ang II ↑→miR-30a ↓→*beclin-1* expression ↑→ excessive autophagy → myocardial hypertrophy.Since myocardial hypertrophy is not a key feature of all stages of cardiac remodeling, this pathway may not apply to all pathophysiological processes that underlie cardiac remodeling.

Mitchell et al. [33], in 2008, were the first to report that miRNAs are present in human plasma in a remarkably stable state, protected from endogenous RNase activity. Cell-free miRNAs are relatively stable due to being packaged inside exosomes, which are 50–90 nm membrane-bound particles that are abundant in plasma. Circulating microRNAs in patients may prove to be a novel class of blood-based biomarker for the diagnosis of diseases. Since it is difficult to obtain samples of cardiac tissue from patients, detecting changes in microRNA expression in peripheral blood may prove to be a potentially more useful approach to acquiring information about the pathophysiological processes underlying heart disease. Numerous studies have now reported the use of microRNA expression in peripheral blood for the diagnosis of cardiovascular diseases [34]–[40]. Since we found that miR-30a is involved in the development of myocardial hypertrophy, we were interested in measuring the changes in miR-30a expression in peripheral blood. The concentration of miR-30a was measured in the plasma of rats from the TAAC group, and patients with LVH. We reported, for the first time, that the plasma concentration of miR-30a increased in rats from the TAAC group, and in patients with LVH. Furthermore, the concentration of miR-30a in plasma was positively associated with IVSd and LVPWd, and use of the plasma level of miR-30a for diagnosis of LVH reached statistical significance. As it was not possible to obtain samples of myocardium from living patients, we were unable to examine the relationship between expression of miR-30a in plasma and its expression in samples of living myocardium. However, the results of our study indicate that miR-30a expression in the circulation has an opposite trend to its expression in cardiac tissue in the TAAC group. We speculate that miR-30a may be transformed from cardiac tissues to the peripheral circulation, and that this process contributes to the down-regulation of miR-30a expression in cardiac tissues of TAAC rats. Testing of this hypothesis deserves further study, as does investigation of the pathway by which miR-30a is transformed from cardiomyocytes to the blood.

In conclusion, miR-30a may be a diagnostic marker for patients with LVH. We hypothesize that myocardial hypertrophy may be improved by supplying ectogenic miR-30a to patients with LVH. If this were shown to be the case, this would provide a novel strategy for the management of LVH and cardiac remodeling.

## References

[1] Dai DF, Johnson SC, Villarin JJ, Chin MT, Nieves-Cintrón M, et al (2011) Mitochondrial oxidative stress mediates angiotensin II-induced cardiac hypertrophy and Galphaq overexpression-induced heart failure. Circ Res 108: 837–846. 21311045 10.1161/CIRCRESAHA.110.232306 PMC3785241

[2] Cao DJ, Wang ZV, Battiprolu PK, Jiang N, Morales CR, et al (2011) Histone deacetylase (HDAC) inhibitors attenuate cardiac hypertrophy by suppressing autophagy.proc natl acad sci USA. Proc Natl Acad Sci U S A 108: 4123–4128. 21367693 10.1073/pnas.1015081108 PMC3053983

[3] Glick D, Barth S, Macleod KF (2010) Autophagy: cellular and molecular mechanisms. J Pathol 221: 3–12. 20225336 10.1002/path.2697 PMC2990190

[4] Nakai A, Yamaguchi O, Takeda T, Higuchi Y, Hikoso S, et al (2007) The role of autophagy in cardiomyocytes in the basal state and in response to hemodynamic stress.Nat Med. 13: 619–624. 10.1038/nm1574 17450150

[5] Zhu H, Tannous P, Johnstone JL, Kong Y, Shelton JM, et al (2007) Cardiac autophagy is a maladaptive response to hemodynamic stress. J Clin Invest 117: 1782–1793. 17607355 10.1172/JCI27523 PMC1890995

[6] Funderburk SF, Wang QJ, Yue Z (2010) The Beclin 1-VPS34 complex–at the crossroads of autophagy and beyond. Trends Cell Biol 20: 355–362. 20356743 10.1016/j.tcb.2010.03.002 PMC3781210

[7] Kang R, Zeh HJ, Lotze MT, Tang D (2011) The Beclin 1 network regulates autophagy and apoptosis. Cell Death Differ 18: 571–580. 21311563 10.1038/cdd.2010.191 PMC3131912

[8] Lu L, Wu W, Yan J, Li X, Yu H, et al (2009) Adriamycin-induced autophagic cardiomyocyte death plays a pathogenic role in a rat model of heart failure. Int J Cardiol 134: 82–90. 18619688 10.1016/j.ijcard.2008.01.043

[9] Zhu H, Wu H, Liu X, Li B, Chen Y, et al (2009) Regulation of autophagy by a beclin 1-targeted microRNA, miR-30a, in cancer cells. Autophagy 2009: 816–823. 10.4161/auto.9064 PMC3669137 19535919

[10] Duisters RF, Tijsen AJ, Schroen B, Leenders JJ, Lentink V, et al.. (2009) miR-133 and miR-30 regulate connective tissue growth factor: implications for a role of microRNAs in myocardial matrix remodeling. Circ Res 104: 170–178, 6p following 178. 10.1161/CIRCRESAHA.108.182535 19096030

[11] Mizushima N, Yoshimori T, Levine B (2010) Methods in mammalian autophagy research. Cell 140: 313–326. 20144757 10.1016/j.cell.2010.01.028 PMC2852113

[12] Fichtlscherer S, De Rosa S, Fox H, Schwietz T, Fischer A, et al (2010) Circulating microRNAs in patients with coronary artery disease.Circ Res. 107: 677–84. 10.1161/CIRCRESAHA.109.215566 20595655

[13] Klionsky DJ, Emr SD (2000) Autophagy as a regulated pathway of cellular degradation. Science 290: 1717–1721. 11099404 10.1126/science.290.5497.1717 PMC2732363

[14] van Rooij E, Olson EN (2007) MicroRNAs: powerful new regulators of heart disease and provocative therapeutic targets. J Clin Invest 117: 2369–2376. 17786230 10.1172/JCI33099 PMC1952642

[15] Gu S, Jin L, Zhang F, Sarnow P, Kay MA (2009) Biological basis for restriction of microRNA targets to the 3′ untranslated region in mammalian mRNAs.Nat Struct Mol Biol. 16: 144–150. 10.1038/nsmb.1552 PMC2713750 19182800

[16] Mellios N, Huang HS, Grigorenko A, Rogaev E, Akbarian S (2008) A set of differentially expressed miRNAs, including miR-30a-5p, act as post-transcriptional inhibitors of BDNF in prefrontal cortex. Hum Mol Genet 17: 3030–3042. 18632683 10.1093/hmg/ddn201 PMC2722882

[17] Mellios N, Galdzicka M, Ginns E, Baker SP, Rogaev E, et al (2012) Gender-Specific Reduction of Estrogen-Sensitive Small RNA, miR-30b, in Subjects With Schizophrenia. Schizophr Bull 38: 433–443. 20732949 10.1093/schbul/sbq091 PMC3329977

[18] Xu Y, Li F, Zhang B, Zhang K, Zhang F, et al (2010) MicroRNAs and target site screening reveals a pre-microRNA-30e variant associated with schizophrenia. Schizophr Res 119: 219–227. 20347265 10.1016/j.schres.2010.02.1070

[19] Kuokkanen S, Chen B, Ojalvo L, Benard L, Santoro N, et al (2010) Genomic profiling of microRNAs and messenger RNAs reveals hormonal regulation in microRNA expression in human endometrium.Biol Reprod. 82: 791–801. 10.1095/biolreprod.109.081059 PMC2842492 19864316

[20] Zaragosi LE, Wdziekonski B, Brigand KL, Villageois P, Mari B, et al (2011) Small RNA sequencing reveals miR-642a-3p as a novel adipocyte-specific microRNA and miR-30 as a key regulator of human adipogenesis.Genome Bioll. 12: R64. 10.1186/gb-2011-12-7-r64 PMC3218826 21767385

[21] Chartoumpekis DV, Zaravinos A, Ziros PG, Iskrenova RP, Psyrogiannis AI, et al (2012) Differential Expression of MicroRNAs in Adipose Tissue after Long-Term High-Fat Diet-Induced Obesity in Mice. PLoS One 7: e34872. 22496873 10.1371/journal.pone.0034872 PMC3319598

[22] Martinez I, Cazalla D, Almstead LL, Steitz JA, DiMaio D (2011) miR-29 and miR-30 regulate B-Myb expression during cellular senescence. Proc Natl Acad Sci U S A 108: 522–527. 21187425 10.1073/pnas.1017346108 PMC3021067

[23] Caruso P, MacLean MR, Khanin R, McClure J, Soon E, et al (2010) Dynamic changes in lung microRNA profiles during the development of pulmonary hypertension due to chronic hypoxia and monocrotaline. Arterioscler Thromb Vasc Biol 30: 716–723. 20110569 10.1161/ATVBAHA.109.202028

[24] Scagnolari C, Zingariello P, Vecchiet J, Selvaggi C, Racciatti D, et al (2010) Differential expression of interferon-induced microRNAs in patients with chronic hepatitis C virus infection treated with pegylated interferon alpha. Virol J 7: 311. 21070682 10.1186/1743-422X-7-311 PMC2996368

[25] Lin J, Lwin T, Zhao JJ, Tam W, Choi YS, et al (2011) Follicular dendritic cell-induced microRNA-mediated upregulation of PRDM1 and downregulation of BCL-6 in non-Hodgkin’s B-cell lymphomas. Leukemia 25: 145–152. 20966935 10.1038/leu.2010.230 PMC3083119

[26] Yu Y, Yang L, Zhao M, Zhu S, Kang R, et al (2012) Targeting microRNA-30a-mediated autophagy enhances imatinib activity against human chronic myeloid leukemia cells. Leukemia 26: 1752–1760. 22395361 10.1038/leu.2012.65

[27] Zhong X, Li N, Liang S, Huang Q, Coukos G, et al (2010) Identification of microRNAs regulating reprogramming factor LIN28 in embryonic stem cells and cancer cells. J Biol Chem 285: 41961–41971. 20947512 10.1074/jbc.M110.169607 PMC3009922

[28] Agrawal R, Tran U, Wessely O (2009) The miR-30 miRNA family regulates Xenopus pronephros development and targets the transcription factor Xlim1/Lhx1. Development 136: 3927–3936. 19906860 10.1242/dev.037432 PMC2778741

[29] Wu T, Zhou H, Hong Y, Li J, Jiang X, et al (2012) miR-30 family members negatively regulate osteoblast differentiation.J Biol Chem. 287: 7503–7511. 10.1074/jbc.M111.292722 PMC3293535 22253433

[30] Gradus B, Alon I, Hornstein E (2011) miRNAs control tracheal chondrocyte differentiation. Dev Biol 360: 58–65. 21945074 10.1016/j.ydbio.2011.09.002

[31] Hand NJ, Master ZR, Eauclaire SF, Weinblatt DE, Matthews RP, et al (2009) The microRNA-30 family is required for vertebrate hepatobiliary development. Gastroenterology 136: 1081–1090. 19185580 10.1053/j.gastro.2008.12.006 PMC2672911

[32] Guo R, Hu N, Kandadi MR, Ren J (2012) Facilitated ethanol metabolism promotes cardiomyocyte contractile dysfunction through autophagy in murine hearts. Autophagy 8: 593–608. 22441020 10.4161/auto.18997 PMC3405837

[33] Mitchell PS, Parkin RK, Kroh EM, Fritz BR, Wyman SK, et al (2008) Circulating microRNAs as stable blood-based markers for cancer detection.Proc Natl Acad Sci U S A. 105: 10513–10518. 10.1073/pnas.0804549105 PMC2492472 18663219

[34] Tijsen AJ, Creemers EE, Moerland PD, de Windt LJ, van der Wal AC, et al (2010) MiR423–5p as a circulating biomarker for heart failure.Circ Res. 106: 1035–1039. 10.1161/CIRCRESAHA.110.218297 20185794

[35] Adachi T, Nakanishi M, Otsuka Y, Nishimura K, Hirokawa G, et al (2010) Plasma microRNA 499 as a biomarker of acute myocardial infarction. Clin Chem 56: 1183–1185. 20395621 10.1373/clinchem.2010.144121

[36] Wang GK, Zhu JQ, Zhang JT, Li Q, Li Y, et al (2010) Circulating microRNA: a novel potential biomarker for early diagnosis of acute myocardial infarction in humans. Eur Heart J 31: 659–666. 20159880 10.1093/eurheartj/ehq013

[37] Fichtlscherer S, De Rosa S, Fox H, Schwietz T, Fischer A, et al (2010) Circulating microRNAs in patients with coronary artery disease. Circ Res 107: 677–684. 20595655 10.1161/CIRCRESAHA.109.215566

[38] Cheng Y, Tan N, Yang J, Liu X, Cao X, et al (2010) A translational study of circulating cell-free microRNA-1 in acute myocardial infarction.Clin Sci (Lond). 119: 87–95. 10.1042/CS20090645 PMC3593815 20218970

[39] D’Alessandra Y, Devanna P, Limana F, Straino S, Di Carlo A, et al (2010) Circulating microRNAs are new and sensitive biomarkers of myocardial infarction.Eur Heart J. 31: 2765–73. 10.1093/eurheartj/ehq167 PMC2980809 20534597

[40] Meder B, Keller A, Vogel B, Haas J, Sedaghat-Hamedani F, et al (2011) MicroRNA signatures in total peripheral blood as novel biomarkers for acute myocardial infarction. Basic Res Cardiol 106(1): 13–23. 20886220 10.1007/s00395-010-0123-2

